# Photocatalytic activity of self-heterojunctioned intermediate phases in HCl protonated and HNO_3_ deconjugated g-C_3_N_4_ nanostructures

**DOI:** 10.1016/j.heliyon.2024.e38025

**Published:** 2024-09-18

**Authors:** S.H. Mousavi-Zadeh, R. Poursalehi, A. Yourdkhani

**Affiliations:** Department of Materials Engineering, Tarbiat Modares University, Tehran, Iran

**Keywords:** Self-heterojunctioned, g-C_3_N_4_, Protonated, Deconjugation, Photocatalysis

## Abstract

This research involved the different acid-treatment conditions of graphitic carbon nitride and its modified nanostructures through thermal polycondensation of urea at various temperatures. X-ray diffraction patterns revealed that processing at a lower temperature than 500 °C resulted in melem and its derivatives, indicating incomplete transformation of urea to g-C_3_N_4_. However, treatment at higher temperatures and the HCl acid treatment led to the formation and expansion of g-C_3_N_4_ networks, as evidenced by notable differences in peak intensities observed in their Fourier-transform infrared and Raman spectra. Scanning electron microscopy analysis illustrated a transition from the granular morphology of melamine to the layered structure characteristic of g-C_3_N_4_. The nanoparticle morphology observed in the HNO_3_ acid treatment sample was attributed to the deconjugation of nanosheets through the highly oxidative acid medium. The most suitable photocatalytic activity for Methylene Blue (MB) degradation under UV and visible light illumination was observed for the samples prepared at 550 °C and HCl post-processed nanostructures. It is proposed that the enhanced photocatalytic activity observed in these samples is most likely attributed to the reduced recombination of photogenerated charge carriers facilitated by heterojunctions formed between different intermediate phases. These findings highlight the potential of modified g-C_3_N_4_ and its derivatives as promising photocatalytic materials for water purification applications.

## Graphical abstract

**Figure:**
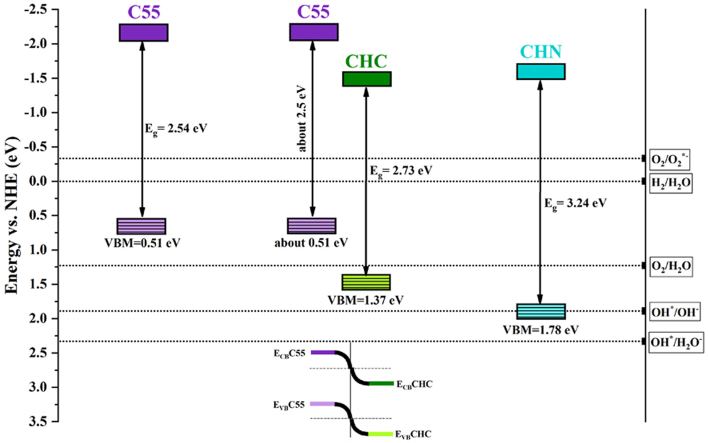

## 1 Introduction

Contemporary research efforts are increasingly focused on addressing critical global issues with a particular emphasis on the energy crisis, climate change, and environmental degradation. Over the past few decades, researchers have been working on developing environmentally sustainable energy sources and efficient photocatalysts to combat pollution. Photocatalysis shows significant potential in converting pollutants into inert inorganic compounds, capturing light, and utilizing it to drive redox reactions by generating charge carriers within the semiconductor [[1], [2], [3]].

Among the plethora of 2D semiconductor materials, graphitic carbon nitride (g-C_3_N_4_), as a metal-free photocatalyst, has emerged as a focal point in the realm of photocatalysis due to its exceptional chemical and thermal stability, facile synthesis and modification methods, and utilization of readily available precursor materials. Graphitic carbon nitride exhibits remarkable efficiency in degrading pollutants under photo-induced processes, owing to its appealing and distinctive electronic band structure [4,5].

Characterized as an indirect bandgap semiconductor with an energy gap of 2.48–2.7 eV, g-C_3_N_4_ can absorb a portion of solar radiation within the visible spectrum. However, a drawback lies in the less positive valence band edge potential of bulk g-C_3_N_4_ (+1.4 V vs. NHE), resulting in moderate photooxidation activity of the holes within its valence band [9]. This limitation is further underscored by the fact that the valence band edge potential of g-C_3_N_4_ falls below the redox potentials of crucial species such as OH^−^/^•^OH or H_2_O/^•^OH (+2.40 V and +2.72 V vs. NHE, respectively), which means hydroxyl radicals cannot be generated directly by holes. Addressing this shortfall is imperative to unlock the full potential of g-C_3_N_4_ in organic pollutant photodegradation [[6], [7], [8]]. Moreover, the two-dimensional planar structure and aromatic ring configuration of g-C_3_N_4_ pose challenges, leading to 3D-stacked layers and poor dispersion in aqueous reaction media. Consequently, catalytic activity may be compromised due to inefficient light absorption and inadequate interaction of reactants [9,10]. In the last decades, diverse strategies have been explored to overcome these challenges and enhance the photocatalytic activity of g-C_3_N_4_. These strategies include protonation, copolymerization, integration with metal oxide semiconductors to form heterostructures, metallic and non-metallic elemental doping, morphology regulation, surface area enhancement, and combined modification techniques [[11], [12], [13], [14]].

An array of preparation and acid-treatment techniques has been explored to enhance photo-induced charge separation and transformation efficiency, along with optimizing light-harvesting capabilities [[15], [16], [17], [18]]. Various chemical agents, including acetic acid, sulfuric acid, hydrochloric acid, and nitric acid, have been employed to alter the characteristics of g-C_3_N_4_, resulting in chemical functionalization and the exfoliation of nanosheets. These approaches effectively modify the chemical and physical properties of g-C_3_N_4_ through acid treatment, leading to an increased number of active sites, changes in optical absorption, improved separation efficiency, and enhanced surface area. The acid-treated two-dimensional g-C_3_N_4_ nanosheets, featuring high porosity and a twisted-conjugated surface, exhibit a broadened bandgap, effective interfacial charge transfer, and prolonged lifetime of photogenerated charge carriers attributed to multiple light scattering effects and the quantum confinement effect. These attributions signify the potential of acid-treated g-C_3_N_4_ nanosheets for highly efficient wastewater purification, as highlighted in Table 1 [19,20].

**Table 1:** Comparing the g-C_3_N_4_ composites reported in this work and the literature reported similar ones in photodegradation.

**Ref. Number**	**Synthesized Material(s)**	**Synthesis Method(s)/Treatment(s)**	**Improvement(s)**	**Application**	**Efficiency**
Ref. [12]	W_18_O_49_/g-C_3_N_4_ Composites	Wcl_6_ Solvothermal/Two-Step Polymerization of Melamine	Extended Photo-Absorption in Visible to Near-Infrared Region/Higher Photogenerated Charge Carrier Separation and Transport	H_2_O_2_ Production	5550 μmg^−1^h^−1^
Ref. [30]	Mo-Impregnated 2D Defective g-C_3_N_4_	Hydrochloric Acid Treatment/Potassium Ion-Assisted Polymerization of Melamine	Larger Pore Volume/Surface Area/Electron-Trapping Ability	Tetracycline(TTC) Degradation	86.6 % within 120 min
Ref. [15]	g-C_3_N_4_/Pt	One-Step and Two-Step Thermal-Induced Polymerization/Sonication	Surface States/Amorphous/Crystalline Homojunctions/Noble Metal Loading	H_2_ Evolutio	4892 μmg^−1^h^−1^
Ref. [29]	Acid-Etched Template Free g-C_3_N_4_	Thermal Condensation of Melamine/Acid Treatment (H_2_SO_4_ and HNO_3_)	Large Specific Surface Area/High Positive Zeta Potential/Reduced Bandgap/Lower Electron-Hole Recombination Rate	MB, Rhodamine B(RhB), Methyl Orange (MO), and Congo Red	79.82 % MB Degradation within 180 min
Ref. [28]	Acid- and Base-Modified g-C_3_N_4_	H_2_SO_4_ Acid and KOH Base Treatment after Polymerization of Melamine	Defect-Induced Structures/OH Group Introduction/Larger Surface Area/More Abundant Pore Structures/Wider Visible Light Absorption Range/Higher Energy Gap Values/Stronger Capacity for Electron-Hole Pair Separation	Cr(VI) Reduction	Almost Converted to Cr(III) after 60 min
Ref. [27]	g-C_3_N_4_ Nanosheets/Cu-tio_2_ Nanofilms Coated with g-C_3_N_4_ Heterostructures	Acetic Acid Treatment of Bulk g-C_3_N_4_ after Polymerization of Melamine/Hydrothermal Process	Successful Separation of Charge Carriers	H_2_ Evolution	261 μmg^−1^h^−1^
Ref. [26]	Exfoliated and Plicated g-C_3_N_4_ Nanosheets	Mixture of Urea and Thiourea Polymerization/HF Acid Treatment (Hydrothermal)	Disordered Defects on Surface/Porous Nanosheets/Increased Number of Active Sites/Enhanced Charge Separation	MB, RhB, MO, and Acid Orange Degradation	75.7 % MB Degradation within 60 min
Ref. [25]	Functionalized Porous g-C_3_N_4_	Oxalic Acid in One-Pot Thermal Polycondensation of Melamine	Highly Porous/Enhanced Surface Area/Abundant Amino Groups Strengthening Visible Light Absorption/Many Active Sites	TTC Degradation	92 % within 90 min
Ref. [24]	P-doped g-C_3_N_4_/α-Bi_2_O_3_ Nanocomposite	Hydrothermal-Calcination/Polycondensation of Melamine and Phytic Acid	Enhanced Light-Harvesting Ability/Heterojunction Formation/Photogenerated Charge Carrier Separation and Transfer	Benzophenones Degradation	94–95 % within 150 min
This work	g-C_3_N_4_ Modified Nanosheets	Urea Polycondensation	Charge Carriers Separation/Surface Modification/Hydrophilicity	MB Degradation	98 % within 120 min

It is not to say that no experimental research has focused on hydrochloric and nitric acid treatments of g-C_3_N_4_, but the fact that in-plane charge transport dominates the overall charge transfer/separation and photocatalytic activity rather than interlayer in graphitic carbon nitride nanostructures have still been suffering from the lack of clarity [21,22]. Also, conventional acid treatment modifications remain the subject of intense debate due to the fact that using strong oxidative acids could lead to the deconjugation of g-C_3_N_4_ layers, resulting in the formation of a barrier for charge transfer through in-plane direction [23].

The primary objective of this research is to investigate the potential impact of conventional acid-treatment modifications on C_3_N_4_ formations. There has been extensive debate surrounding the use of strong oxidative acids due to the potential deconjugation of g-C_3_N_4_ layers, which may lead to the development of a barrier for charge carrier transport through the in-plane direction and the occurrence of detrimental electron-hole recombination phenomena. The ability to comprehend the mechanisms through which acid treatments may influence the properties of g-C_3_N_4_ is essential for advancing our knowledge in this research area. Moreover, it is crucial to identify the precise conditions under which these modifications result in favorable or detrimental outcomes in order to inform future research and potential applications of g-C_3_N_4_. The incidental objective of this research aims to elucidate the pathways of g-C_3_N_4_ nanosheet formation through urea polycondensation without acid treatment, as well as to enhance the photocatalytic activity of carbon nitride structures through acid treatment. The thermal condensation of urea between 400 °C and 550 °C leads to the formation of graphitic carbon nitride, as evidenced by various analyses. X-ray diffraction analysis indicated that processing at a lower temperature than 500 °C resulted in the presence of melem and its derivatives, signifying incomplete transformation of melamine to g-C_3_N_4_. Acid treatment of the synthesized sample at 550 °C with hydrochloric acid improved the catalytic surface properties by facilitating the exfoliation of the 3D-stacked layers. Conversely, treatment with nitric acid increases optical bandgap and in-plane deconjugation, consequently reducing the photocatalytic activity. The sample synthesized at 550 °C and treated with hydrochloric acid exhibited the highest photocatalytic activity, possibly due to the formation of self-assembled g-C_3_N_4_ heterostructures with its intermediate phases during thermal polycondensation and acid treatment. These findings underscore the potential of acid treatment for g-C_3_N_4_ and its derivatives as promising photocatalytic materials for water purification applications.

## 2 Experimental

### 2.1 Materials

Urea (CO(NH_2_)_2_), ≥99 % Merck), nitric acid (HNO_3_, ≥65 % Merck), hydrochloric acid (HCl, ≥37 % Merck), ethanol (C_2_H_5_OH, ≥99.7 % Merck), deionized water with σ = 0.2 μS/cm, Isopropanol ((CH_3_)_2_CHOH, IPA, Dr. Mojallali) ethylenediaminetetracetic acid disodium (C_10_H_14_N_2_Na_2_O_8_, EDTA-2Na, Cinagen), benzoquinone (C_6_H_4_O_2_, BQ, Sigma-Aldrich) were used without additional treatment.

### 2.2 Preparation of g-C_3_N_4_

**Scheme 1:**
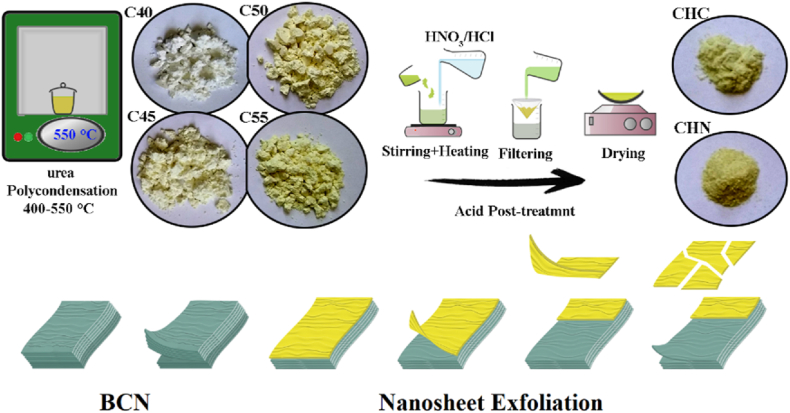
The preparation method and two different approaches for modification of graphitic carbon nitride photocatalyst.

Bulk graphitic carbon nitride (BCN) powder was synthesized following a previously established protocol as shown in Scheme 1. Initially, 15 g of urea were placed in a covered ceramic crucible and subjected to heating in a muffle furnace within 400–550 °C temperature range. The temperature was incrementally raised at a rate of 10 °C/min until reaching the final temperature, where it was maintained for 4 h in air. Subsequently, the crucible was allowed to cool to room temperature, resulting in stacked layers of g-C_3_N_4_ powder with varying yield efficacy depending on the heating condition. The products were designated as C40-C55. The yield of g-C_3_N_4_ was determined to be 31.4 %, 20.2 %, 15.9 %, and 12.2 % for C40, C45, C50, and C55, respectively.

### 2.3 Protonation and exfoliation of g-C_3_N_4_

The exfoliation of C55 nanopowders, as a more rational candidate for photocatalysis, was performed in order to synthesize HCl acid-treated carbon nitride. Approximately 100 mg of C55 was added to 50 mL of HCl solution, following vigorous stirring in a baker. The resulting solution was then transferred to a 100 mL Teflon-lined stainless steel autoclave, heated at 120 °C for 2 h, and subsequently allowed to cool to room temperature. Following solvent evaporation, photocatalyst powder was collected by washing with deionized water three times and centrifuged at 4000 rpm. The resulting powder was air-dried at 80 °C for 24 h, yielding sample named CHC. The process of g-C_3_N_4_ protonating with hydrochloric acid was observed to enhance surface functionalization, resulting in improved water dispersibility and hydrophilicity.

### 2.4 Deconjugation and re-polycondensation of g-C_3_N_4_

In another approach, 3 g of C55 powder was refluxed in 50 mL of concentrated HNO_3_ solution at 80 °C for 3 h to produce a stable colloidal solution. The colloid was then filtered and aged for 48 h, after which the white powder was repolymerized by heating at 350 °C in a closed ceramic crucible to complete the removal of HNO_3_, owing to the reversible nature of hydrogen bond formation and the soft polymeric matrix. The resulting agglomerated powder, designated as CHN, was pulverized into powder using a pestle and mortar. The strongly oxidizing properties of ^−^NO_3_ in HNO_3_ played a significant role in the sol processing of g-C_3_N_4_ structures, triggering depolymerization of g-C_3_N_4_ nanosheets, unlike HCl, which primarily surface-functionalizes the material. Previous reports have highlighted the role of HNO_3_ in breaking structural hydrogen bonds between melon units and making the subdivision of g-C_3_N_4_ structures to its building blocks easier [23].

## 3 Characterizations

The comprehensive characterization techniques were employed to investigate various aspects of the synthesized materials, including structural, morphological, thermal, compositional, and optical properties. Morphologies were investigated using Field-Emission Scanning Electron Microscopy (FE-SEM), employing a TESCAN MIRAIII. Elemental Analyses of carbon and nitrogen contents were analyzed using a MIRAII TESCAN-SAMX. Nitrogen Adsorption-Desorption Isotherms was acquired at 77 K using a BELSORP MiniII surface area and porosity analyzer, following a 14-h outgassing period at 120 °C before measurements. X-ray Diffraction (XRD) Analysis was conducted using a PXRD X'Pert MPD, Philips X-ray powder diffractometer with Cu Kα radiation λ = 1.5406 Å in the range of 10–80° for phase identification. Thermal Gravimetric Analysis (TG/DTA) was performed using a Mettler Toledo (TGA/DSC 1) thermal analyzer. X-ray Photoelectron Spectroscopy (XPS) measurements were conducted using a BESTEC (EA10) dual Mg–Al anode X-ray source instrument, with the shift of binding energy calibrated using an internal standard of C1s level at 284.8 eV. Fourier Transform Infrared (FTIR) Spectra was carried out in transmission mode over the range of 4000-400 cm^−1^ using a PerkinElmer Spectrum RXI spectrometer with KBr discs. Raman Spectra were obtained with a Takram P50C0R10 spectrometer at an excitation wavelength of 752 nm. UV–visible transmittance spectra were recorded utilizing a SCO-TECSP UV-26 spectrometer between 200 and 850 nm.

## 4 Adsorption and photocatalytic activity measurement

The assessment of photocatalytic efficacy involved the degradation of MB as a conventional model pollutant under visible light illumination. Visible light was sourced from a 15 W white LED equipped with a 400 nm cut-off filter to ensure the provision of visible light and a 15 W UV LED equipped for more irradiation sensitivity investigation. For photocatalytic activity test, 12.5 mg of photocatalysts were dispersed in a 50 mL solution containing 5 mg L⁻^1^ MB under magnetic stirring for photocatalytic evaluation. Prior to light exposure, the dispersion underwent a 60-minute dark incubation period under continuous magnetic stirring to establish adsorption-desorption equilibrium between the catalyst and the dye molecules. Upon light irradiation, samples were collected at 30-minute intervals. Following centrifugation of the catalyst, the remaining solution was analyzed using a UV–visible spectrometer. For comparative analysis, photodegradation reactions were conducted in the presence of g-C_3_N_4_ and without any catalyst. Degradation efficiency was quantified by calculating the ratio of C to C_0_, where C represents the concentration of the remaining MB solution at each irradiation time, and C_0_ denotes the initial MB concentration. This approach provided a quantitative measure of the photocatalytic performance of the synthesized samples in degrading the MB dye under visible light illumination.

## 5 Results and discussion

### 5.1 Formation of g-C_3_N_4_ nanosheets

**Fig. 1:**
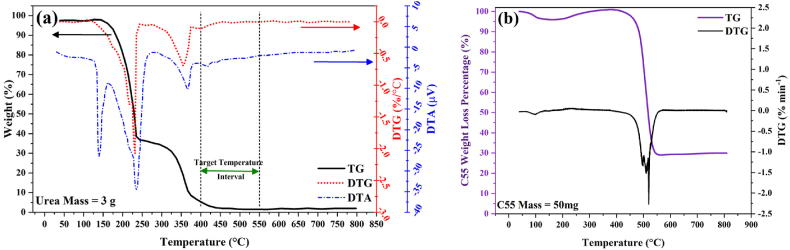
Thermogravimetric analysis of (a) urea, and (b) C55 sample.

**Fig. 2:**
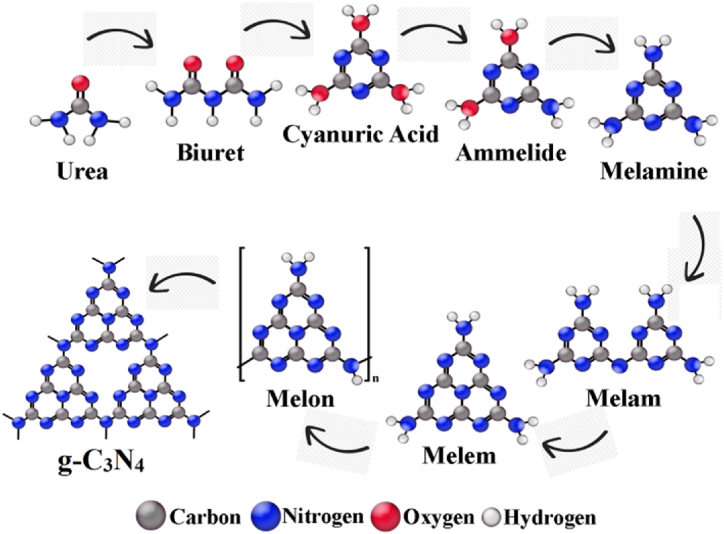
Possible phase transformation pathway of urea to heptazine-based graphitic carbon nitride nanosheets.

It is established that when subjected to heat, urea undergoes condensation reactions accompanied by the elimination of ammonia or deamination. Consequently, a thermal analysis of melamine was imperative to explore alterations in its physical and chemical states relative to increasing temperature. The experimental measurements were conducted and the outcomes are depicted in Fig. 1, Fig. 2. Thermogravimetric analysis is a method that primarily observes changes in the mass of a substance as a function of temperature. In our study, the TG curve exhibits a significant transition from 254 °C to 367 °C, followed by a smaller transition from 367 °C to 498 °C. These transitions signify the evaporation of ammonia and subsequent total mass loss. Correspondingly, the derivative thermogravimetric analysis curve displays two distinct peaks in the same temperature regions along with their respective intensities. The initial and more pronounced peak denotes a rapid deamination of urea and a substantial weight reduction, while the secondary and less pronounced peak corresponds to further condensation processes leading to the formation of melem (2,5,8-triamino-tri-striazine) and its derivatives [23]. Subsequently, a third transition initiates after about 500 °C, attributed to the conversion of melem derivatives into g-C_3_N_4_ accompanied by additional mass loss. Differential scanning calorimetry offers insights into thermal changes without affecting the sample mass. Thus, the prominent peak at about 360 °C indicates an endothermic event attributed to the melting of melamine. The thermal analysis of urea identifies three temperature regions of interest including 100–175 °C, 175–250 °C, and exceeding 350 °C. The polycondensation of urea appears to initiate at 400 °C, with a partial thermal condensation of g-C_3_N_4_ occurring within the 400–500 °C temperature range. Subsequently, the evaporation of g-C_3_N_4_ nanosheets at approximately 600 °C results in a higher mass yield for the C55 sample compared to g-C_3_N_4_ nanosheets prepared at higher temperatures, confirmed by other works [[31], [32], [33]]. Consequently, the temperature range of 400–550 °C presents a promising window for investigating the transformation of urea into g-C_3_N_4_ nanosheets and the generation of self-heterostructured g-C_3_N_4_ and its derivatives.

### 5.2 Morphology, BET surface, and elemental analysis

**Fig. 3:**
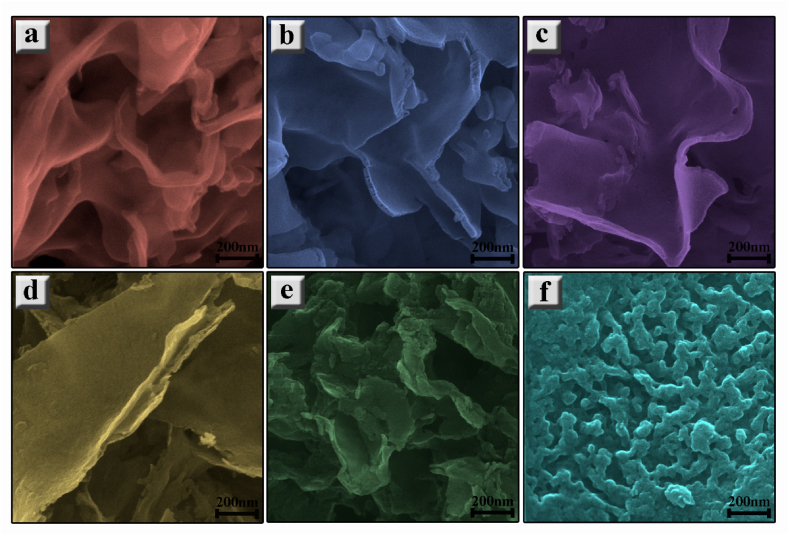
Morphology of (a) C40, (b) C45, (c) C50, (d)C55, (e) CHC, and (f) CHN carbon nitride nanostructures.

**Fig. 4:**
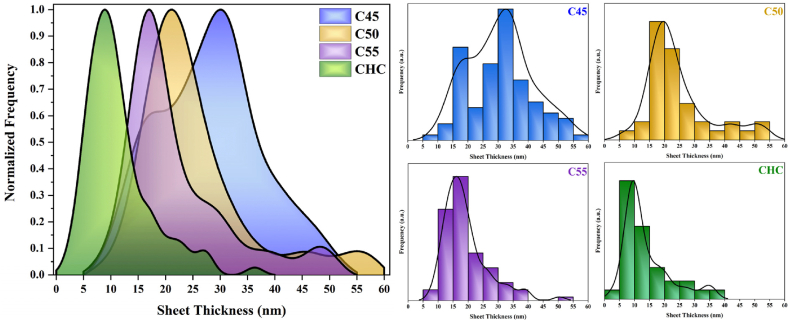
Sheet thickness distribution of carbon nitride nanostructures.

The FE-SEM images of graphitic carbon nitride nanostructures in Fig. 3 reveal the layered morphology of all samples except for CHN. 3D stacked-layered sheets are observed with thicknesses ranging from 5 nm to 60 nm, as shown in Fig. 4. Particularly noteworthy is the appearance of tubular structures in the C40 sample, a common phenomenon in the initial stages of lamellar g-C_3_N_4_ formation. This occurrence is attributed to the release of NH_3_ during the polycondensation process. As NH_3_ traverses through moderately packed melamine layers, it contributes to the formation of slightly rolled g-C_3_N_4_ sheets. At 550 °C, a more organized nanosheet morphology is evident, with decreased thickness [34]. The C55 sample exhibits disjunction and fragmented layers at the edges, showcasing sublayers with a thickness of approximately 15 nm, which are more pronounced in this sample. The transition from granular to layered morphology correlates with the temperature of the polycondensation process, wherein elevated temperatures facilitate increased condensation degree. Despite the layered characteristic of g-C_3_N_4_ structures being preserved, some nanosized pores are periodically distributed, especially in the case of the CHC post-processing condition. Conversely, subjecting the material to the more reductive HNO_3_ acid-treatment process results in an irregular morphology featuring small agglomerated nanoparticles of g-C_3_N_4_ [35].

**Fig. 5:**
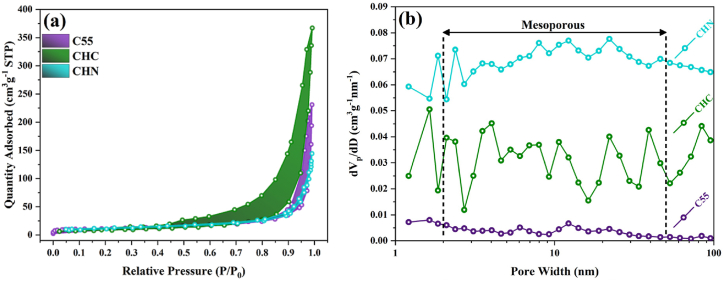
(a) Adsorption-Desorption Isotherm and (b) pore diameter distribution for C55, CHC, and CHN nanostructures.

The N_2_ adsorption-desorption isotherms and Barrett–Joyner–Halenda pore-size distributions presented in Fig. 5 demonstrate characteristic type IV isotherms, a signature of mesoporous materials across all samples. Notably, the pore-size distribution curves reveal broadened features around ∼200 Å (diameter of 40 nm), signifying the mesoporous nature of the samples. Furthermore, the CHC sample exhibits a broader distribution encompassing large mesopores and small macropores at 100–1000 Å (diameter between 20 and 200 nm). Correspondingly, the BET surface area values, outlined in Table 2, escalate from ∼37–47 m^2^g^-1^ with the HCl acid treatment process. Materials characterized by high surface area and large pore size are anticipated to exhibit enhanced photocatalytic properties owing to improved reactant adsorption and mass transfer capabilities.

**Table 2:** BET surface area and average pore size of carbon nitride nanostructures.

Sample	BET Surface Area (m^2^g^−1^)	Average Pore Size (nm)
C55	37	23
CHC	47	73
CHN	30	32

**Fig. 6:**
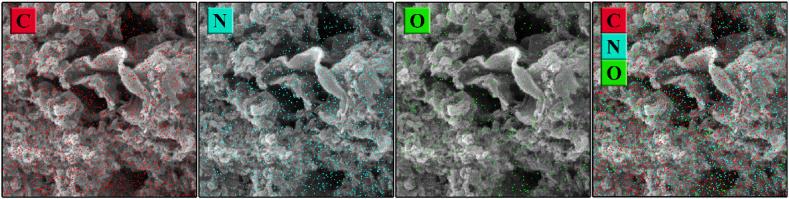
Energy-dispersive X-ray elemental mapping of C55 sample.

**Fig. 7:**
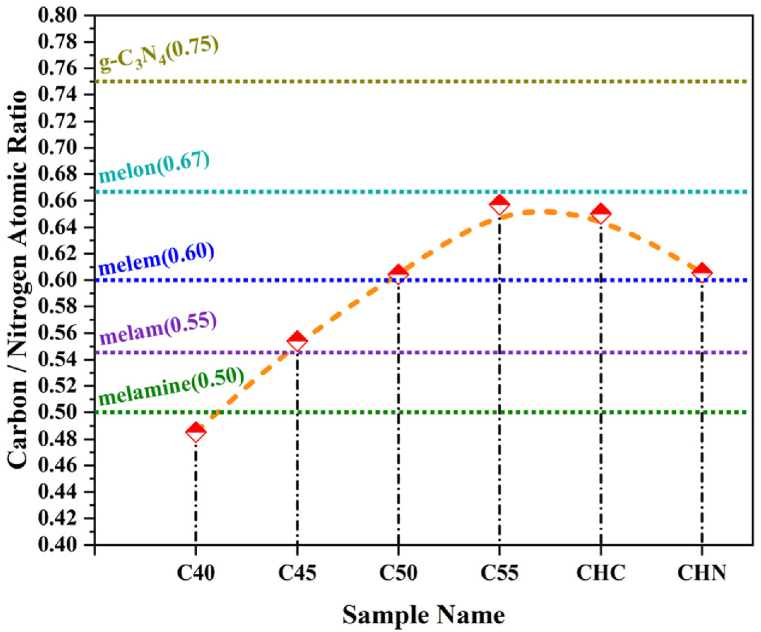
Dependency of C/N atomic ratio to the condensation temperature and acid modification condition.

EDS maps of g-C_3_N_4_ samples are shown in Fig. 6 and Table 3. The C/N ratio of samples increases as the annealing temperature rises to 550 °C as depicted in Fig. 7. Both C55 and Acid-treated CHC samples exhibited the highest melon-based C/N ratio of 0.59, comparable to the melamine-like C/N ratio in C40(0.5), which deviates from the optimal ratio expected for g-C_3_N_4_ (0.75). This discrepancy may be attributed to the prevalence of amino groups or carbon vacancies within the structure. Conversely, the CHN sample demonstrated a lower C/N ratio of 0.6 and elevated presence of oxygen atoms, signifying the oxidation process within the composition of material [36,37].

**Table 3:** EDS elemental analysis of CN nanostructures prepared in different annealing condition.

Sample	C (At%)	N (At%)	O (At%)	C/N Atomic Ratio
C40	32.65	67.26	0.09	0.485
C45	35.25	63.61	1.14	0.554
C50	36.24	59.96	3.80	0.604
C55	38.99	59.34	1.67	0.657
CHC	37.98	58.41	3.61	0.650
CHN	33.92	56.02	10.06	0.606

### 5.3 XRD patterns and structural properties

**Fig. 8:**
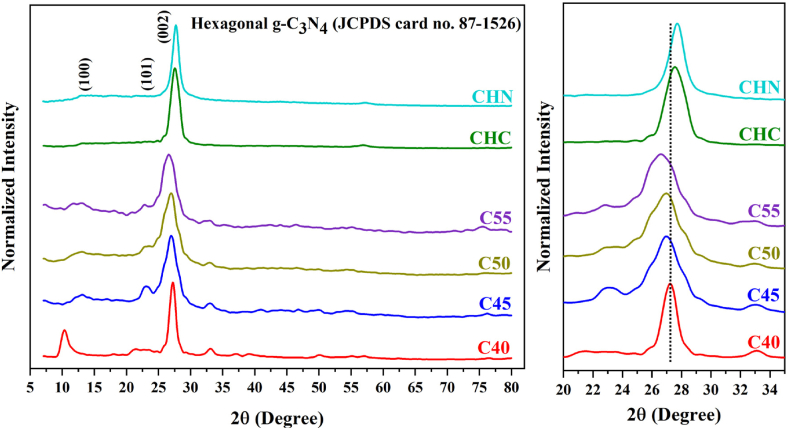
XRD patterns of CN nanostructures at different conditions, and (002) peak shift.

The X-ray Diffraction patterns of synthesized CN structures are presented in Fig. 8. A sequence of diffraction peaks is discernible for sample C40 within the 2θ range spanning from 10.6° to 32.4°. Notably, several of these peaks (at 10.6°, 19.7°, 21.4°, 30.7°, and 32.4°) can be attributed to melamine, cyanuric acid, melem and its derivatives like dimelem, consistent with thermal analysis findings. However, these phases serve as intermediates in the polycondensation process from urea to g-C_3_N_4_, indicating an ongoing incomplete transformation [[38], [39], [40]]. For sample C55, two distinct peaks characteristic of g-C_3_N_4_ are observable. One is the prominent peak at 26.6° corresponding to the (002) plane, indicative of interlayer stacking of aromatic segments with a distance d = 0.330 nm related to hexagonal g-C_3_N_4_ (JCPDS Card no. 87–1526). This stacking configuration is denser compared to carbon graphene units and crystalline graphite packing. The heightened packing density perpendicular to the layers in aromatic systems with vacancy substitution can be attributed to electron localization and stronger interlayer binding. The other is the faint peak at 13.1° corresponding to the (100) plane, related to the in-plane structural arrangement of tri-s-triazine units with an interplanar distance of d = 0.680 nm [41]. Regarding C55, both (100) and (002) diffraction peaks exhibit diminished intensity. Additionally, a slight shift of the (002) peak to 2θ = 27.6° indicates an interlayer distance of d = 0.320 nm, suggesting denser-packed layers of CHC and CHN. The XRD patterns imply that intermediates such as melem derivatives may form at approximately 400–450 °C [42,43]. However, extensive polycondensation processes occur above 500 °C, culminating in the development of extended g-C_3_N_4_ networks characterized by a layered structure.

### 5.4 XPS analysis and surface chemistry

**Fig. 9:**
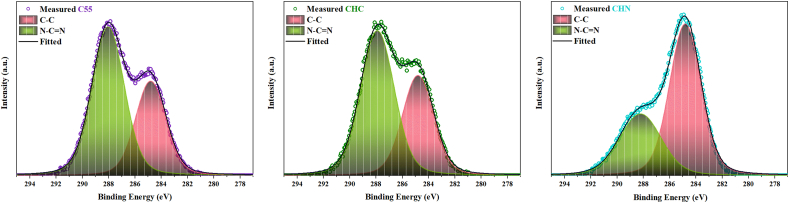
Deconvolution of C 1s peak for C55, CHC, and CHN nanostructures.

**Fig. 10:**
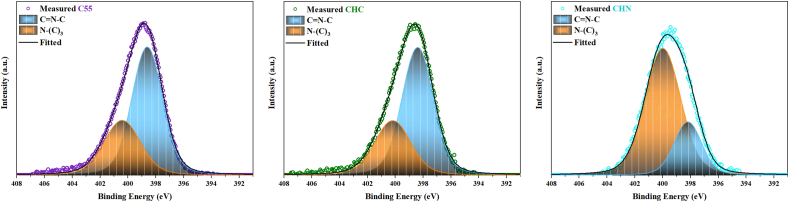
Deconvolution of N 1s peak for C55, CHC, and CHN nanostructures.

**Fig. 11:**
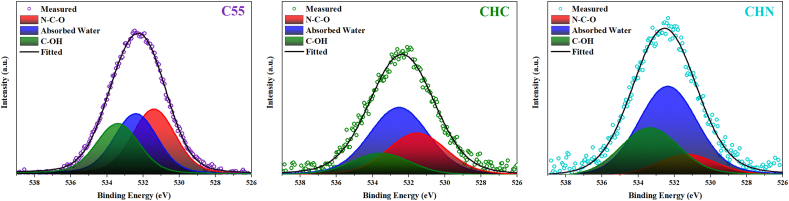
Deconvolution of O 1s peak for C55, CHC, and CHN nanostructures.

**Fig. 12:**
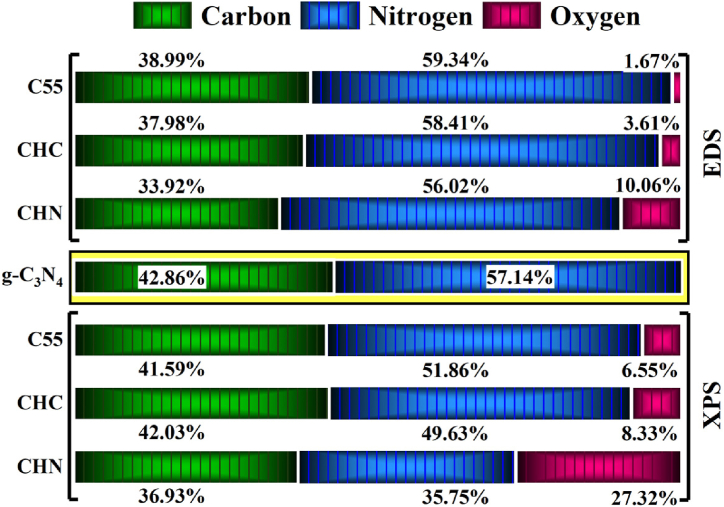
Comparison of elemental contribution of carbon, nitrogen, and oxygen for C55, CHC, and CHN.

**Fig. 13:**
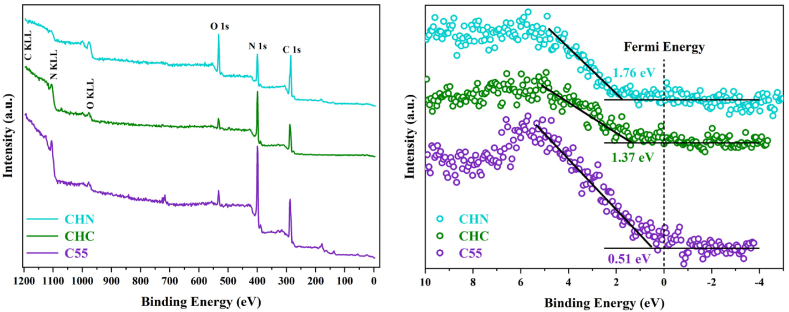
Survey spectra (Left), and valence band edge calculation using low-energy spectra (Right) for C55, CHC, and CHN.

The X-ray photoelectron spectroscopy was carried out to elucidate the elemental composition of the examined C55, CHC, and CHN. The survey scan demonstrates the presence of carbon, nitrogen, and a marginal quantity of oxygen across all samples. The occurrence of oxygen is ascribed to its absorption during the experimental procedure conducted under inert atmospheric conditions or the survey scan. The deconvolution of the C1s spectrum, as depicted in Fig. 9, reveals two indistinguishable peaks. The peak observed at 287.5 eV corresponds to sp2-bonded carbon in the N-C=N group. The peak situated at 284.2 eV is attributed to sp2-bonded carbon within C-C bonds and adventitious hydrocarbons originating from the XPS instrument. Deconvoluted N 1s and O 1s spectra are depicted in Fig. 10 and Fig. 11 respectively. In Fig. 10, the N 1s spectra exhibit three distinct peaks upon fitting. The principal peak, centered at 398.0 eV, is associated with sp2-hybridized nitrogen in C=N-C bonds within triazine rings, and the peak at 399.8 eV is assigned to N-(C)_3_ bonds in tertiary nitrogen groups. The relative intensities of the primary peaks suggest a decrease in the proportion of N-(C)_3_ groups relative to C=N-C as the HNO_3_ acid process is accomplished. This observation indicates a lesser incorporation of tri-s-triazine units interconnected by N-(C)_3_ groups, forming smaller g-C_3_N_4_ networks. Interestingly, no meaningful change in this proportion was observed due to the conservation of tri-s-triazine blocks for CHC [[44], [45], [46]]. The carbon-to-nitrogen (C/N) atomic ratio, as delineated in Fig. 12 through elemental analysis, demonstrates a diminution trend with increasing oxidative acid treatment. Regarding EDS analysis, however, the determined C/N ratios in all cases fall below the theoretical value of 0.75. This discrepancy aligns with prior studies, suggesting the persistence of amino groups due to incomplete polycondensation [47]. As depicted in Fig. 13, the valence band energies were calculated based on the XPS survey and using low-energy spectra, which is crucial to reveal the mechanism of photocatalytic processes. The high-resolution XPS spectra in Fig. 11 demonstrate the presence of the O 1s core level at 532.6 eV for CN nanostructures, primarily attributed to absorbed water. Furthermore, two distinct peaks are identified at 531.6 and 533.5 eV, corresponding to N-C-O and C-OH chemical species, respectively [[48], [49], [50]].

### 5.5 FTIR and Raman

**Fig. 14:**
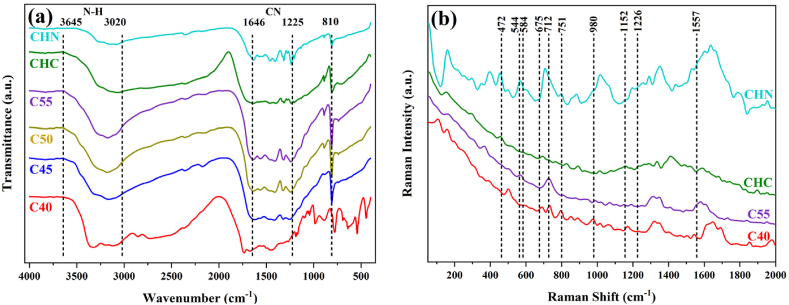
(a) FTIR and (b) Raman Spectra of CN nanostructures.

The chemical bonds and composition of the samples underwent further analysis through FTIR spectroscopy, as illustrated in Fig. 14. All spectra exhibit a distinct absorption peak at 810 cm^−1^, attributed to the breathing mode of triazine units. Notably, a series of peaks spanning from 1225 to 1646 cm^−1^ dominate the spectra, conforming to the C-N heterocycles stretching modes. For C40, two distinctive peaks at 1460 and 1610 cm^−1^ indicate the presence of melem in tri-s-triazine motifs. Additionally, peaks at 1238, 1322, and 1402 cm^−1^ correspond to the aromatic secondary or tertiary amines of C-N bonding, suggesting the formation of dimelem through the connection of two melem molecules. In the cases of C45-CHC samples, the above-mentioned peaks and 1460 cm^−1^ persist but with significantly increased intensity, indicating a higher polycondensation degree, consistent with the XRD results. The intensity of peaks appears to be correlated with the polycondensation degree of tri-s-triazine motifs. Two peaks observed at 1561 and 1640 cm^−1^ are ascribed to the stretch vibrations of asymmetric C-N side-chain. Lastly, the broad peaks within the 3000–3700 cm^−1^ range are assigned to the N-H stretching modes of residual amino groups and the OH bond from water molecules adsorbed on the CN sheets. A significant disparity in peak intensity is observed between melamine-like C40 and acid-post-treated CHN as the NH_2_ groups of C40 undergo decomposition, contributing to the formation of g-C_3_N_4_ networks and inversely-activated decomposition of g-C_3_N_4_ towards dimelem units, respectively [51,52].

The Raman spectra of melamine-like C40, C55, CHC, and CHN samples are illustrated in Fig. 14. The most prominent peak in the melamine Raman spectrum, appearing at 675 cm^−1^, corresponds to the breathing 2 mode of the triazine ring. Another peak at 980 cm^−1^ signifies the breathing 1 mode of the triazine ring. The second ring-valence vibration of species E′ is observed near 1557 cm^−1^, while in the low-frequency region, the peak at 584 cm^−1^ also pertains to species E'. For sample C40, a series of peaks are discernible throughout the spectra, predominantly assignable to melem and its derivatives, corroborating the findings from XRD and FTIR analyses. Specifically, peaks at 544, 751, and 1152 cm^−1^ are attributed to the A1 vibrations of the tri-s-triazine ring. The intensity of the peak at 980 cm^−1^ diminishes as more triazines combine to form the tri-s-triazine motifs of melem for C55. As the acid-treatment process applies, the CHN sample seemingly undergoes a reversely-activated transformation into melems as CHC remains unchanged [53,54]. Several characteristic peaks of g-C_3_N_4_ at 472, 712, 980, and 1226 cm^−1^ are evident in the spectra of all samples. The intensity of peaks at 751 and 1152 cm^−1^ markedly decreases, while the peak at 544 cm^−1^ disappears entirely, indicating the suppression of A1 vibrations due to the further combination of tri-s-triazine units. It should be noted that the multitude of peaks and the presence of melem in the C40 and CHN Raman spectra confirm the incomplete status of the polycondensation process at 400 °C and acid treatment [31,55].

### 5.6 Optical bandgap

**Fig. 15:**
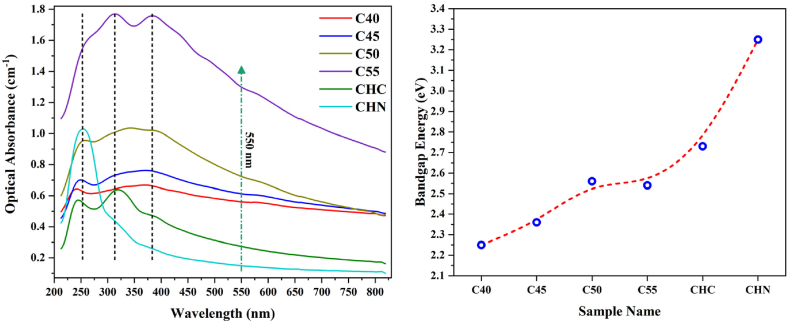
Optical absorbance of nanosheets synthesized at various conditions.

**Fig. 16:**
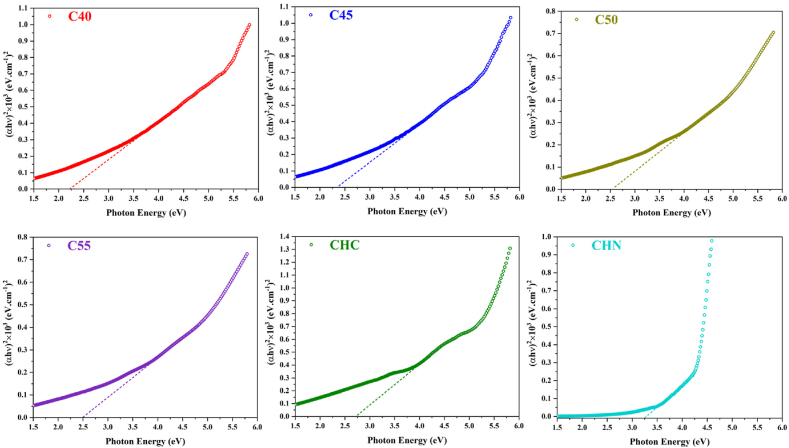
Bandgap estimated by Tauc equations for CN nanostructures.

The optical characteristics of the synthesized samples were assessed through UV–vis transmittance spectroscopy. Fig. 15, Fig. 16 illustrate the results, wherein the bandgap energies of C40-CHN vary from 2.25 eV for C40 to 3.24 eV for CHN, with corresponding absorption edges at about 550 nm and 400 nm, respectively. Notably, the CHC exhibited a moderated bandgap energy of 2.73 eV, correlating to an absorption edge at about 450 nm. As the process temperature increased, a notable trend emerged wherein the optical band edges of the samples shifted towards shorter wavelengths, conforming with a significant increase in the bandgap. This phenomenon can be rationalized by the polymeric network extension of g-C_3_N_4_, facilitated by decreased connectivity of tri-s-triazine units along two dimensions and the resultant 2D quantum confinement at elevated temperatures [56,57]. The structural acid modification of CHC leads to the conservation of C-N clusters, typically associated with a slightly extended optical bandgap. The widening of the bandgap, alongside enhanced light harvesting capacity, theoretically augments the photocatalytic efficacy of g-C_3_N_4_, as suggested in numerous studies [58,59].

### 5.7 Photocatalysis

**Fig. 17:**
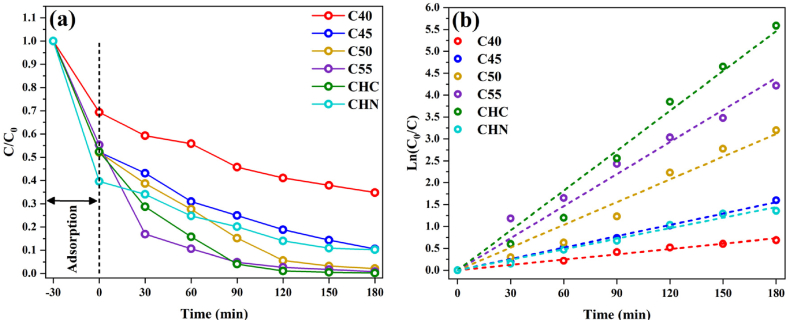
(a) Photocatalytic activity and (b) pseudo-first-order model for CN nanostructures.

**Fig. 18:**
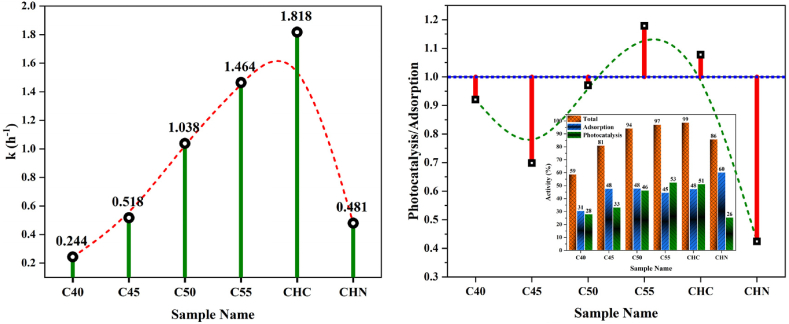
The pseudo-first-order photocatalytic degradation rate constant and photocatalysis/adsorption ratio for CN nanostructures.

The efficacy of g-C_3_N_4_ samples in MB removal under UV and visible light irradiation was evaluated to assess their potential for water purification. The underlying reactions in MB oxidation or reduction were conducted by photogenerated electrons and holes, resulting in the production of non-toxic compounds. Fig. 17 illustrates the temporal variation of MB final concentration during the adsorption and photocatalytic processes as the thermal polycondensation temperature increases from 400 to 550 °C. Previous studies have highlighted that MB concentration does not diminish with light exposure, a notion reaffirmed in our investigation where photocatalytic activity was observed solely under light illumination. The absorption intensity of C55 in the visible-light spectrum exceeded that of C40, C45, and C50, indicating a significant potential for light absorption. C55, moreover, demonstrated superior photocatalytic activity compared to bulk C40, attributed to its larger surface area and mesoporous structure. The exfoliation of g-C_3_N_4_ nanosheets resulted in a high surface area in C55 nanosheets, enhancing its multiple light scattering capacity and providing increased catalytic active sites, thereby contributing to accelerated photocatalysis rates. These findings suggest that the semi-completed polycondensation of urea and positive integrated effects have led to the attractive photocatalytic performance of C55 [60,61]. The pseudo-first-order photocatalytic degradation rate constant and photocatalysis/adsorption ratio for CN nanostructures are depicted in Fig. 18.

**Fig. 19:**
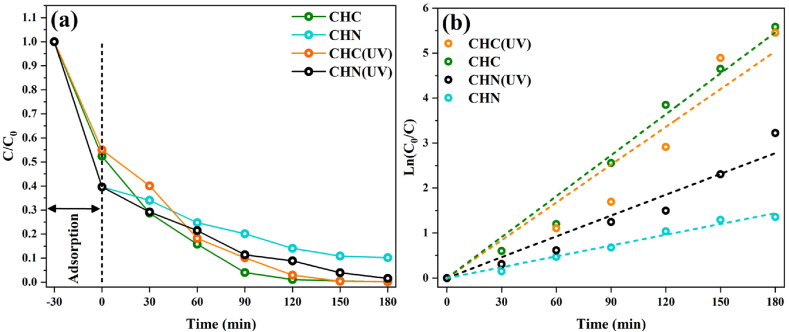
Photocatalytic activity (a) and pseudo-first-order model (b) of CHC and CHN under UV irradiation.

Under visible light irradiation, C55 and CHC samples exhibit superior photocatalytic activity, reducing MB concentration by about 98 % within 120 min. CHN displays a comparable performance, with a reduction of 86 %, while C40 demonstrated the weakest efficacy, achieving only a 59 % degradation. As shown in Fig. 19 (a) and (b), the same trend was remarkably observed under UV irradiation, with the CHN sample showcasing improved efficiency, reducing MB by 98 % in 180 min. Notably, the observed photocatalytic activity directly corresponds to changes in BET-specific surface area, pore size diameter, and bandgap energy variation. The significant influence of surface parameters on photocatalytic performance emerges as a decisive factors in our experimentation. We believe that the enhanced photocatalytic activity of the C55 and CHC samples may be attributed to the lower bandgap and formation of heterojunctions between g-C_3_N_4_ and its derivatives. At 500 °C, the polycondensation and protonation process remains incomplete, leading to the coexistence of intermediate g-C_3_N_4_, melem, and dimelem phases [[62], [63], [64]]. These phases foster the creation of heterojunctions, thereby improving the separation of photogenerated carriers and consequently augmenting photocatalytic activity due to suitable valence band potential [65,66].

**Fig. 20:**
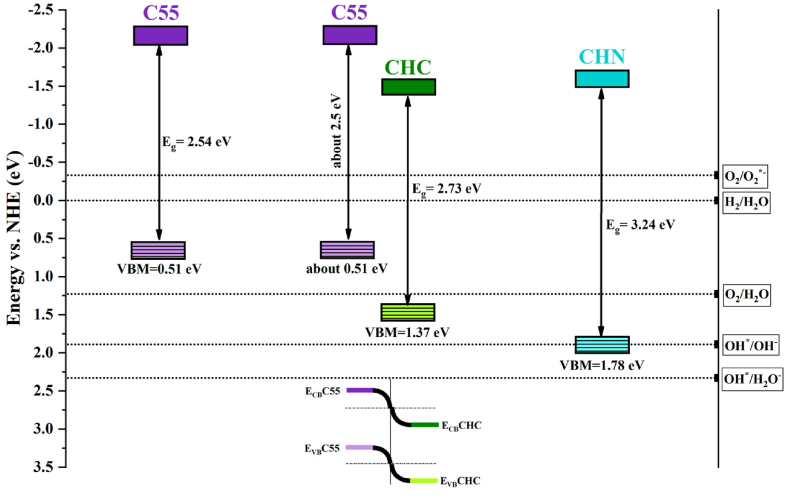
Possible band structure configuration of C55, CHC, and CHN nanostructures.

**Scheme 2:**
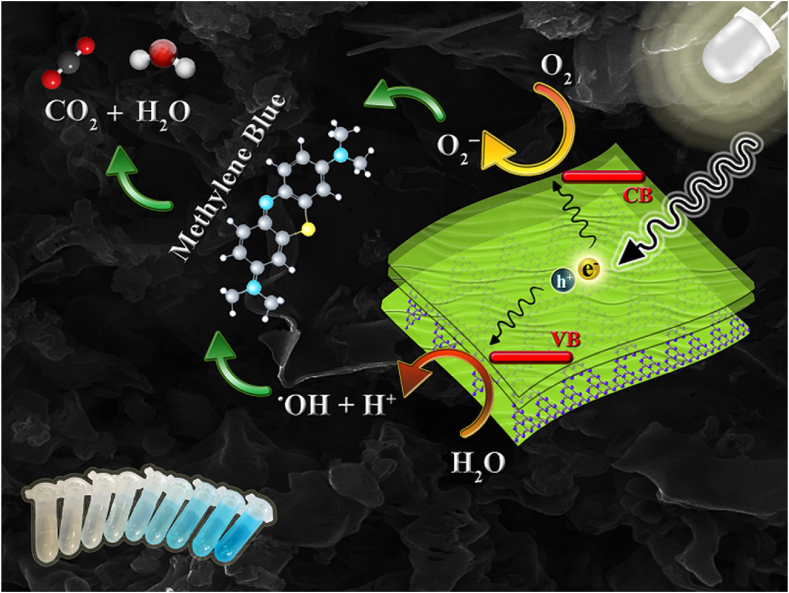
MB degradation on the surface of graphitic carbon nitride nanosheets.

The schematic diagram in Fig. 20 illustrates the potential band structure of C55, CHC, and CHN, which helps to explain the photocatalytic mechanism. Acid-treated nanostructures have a more positive conduction band than C55, indicating a lower reductive ability likely due to the C-O/C-Cl bonds formation. This results in the immobility of π-system electrons instead of σ-system electrons, thus inhibiting the separation of photogenerated charge carriers and accelerating the recombination rate of electron/hole pairs [67]. Valence bands, obtained from low-energy X-ray photoelectron spectroscopy, and conduction bands, calculated from the bandgap energies using the Tauc plot method, show a positive shift in CHC and CHN compared to C55, indicating their increased oxidative ability. Consequently, the photo-excited electrons remaining in the conduction band of CHC can be trapped by O_2_ to yield ^•^O_2_^-^, while the remaining holes have sufficient energy to degrade organic compounds, as shown in Scheme 2. These can facilitate the separation and transfer of photogenerated charges, thereby preventing the recombination of electrons and holes [26]. The higher bandgap energy is an impediment factor in the initial phase of generating photo-induced charge carriers, leading to a proposed mechanism that does not apply to CHN nanostructures, as observed in CHC nanosheets.

### 5.8 Scavenger test

**Fig. 21:**
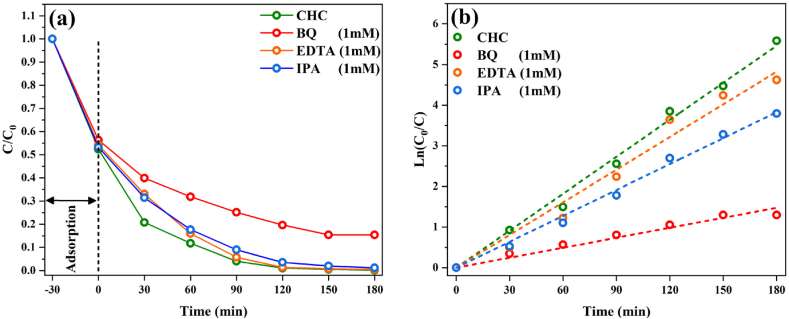
Photocatalytic activity in the presence of scavengers for CHC sample.

In the scavenger test presented in Fig. 21, it is observed that the introduction of BQ led to substantial suppression of the photocatalytic activity for g-C_3_N_4_ in comparison to the scenario with no scavenger addition. This suggests that O_2_^−^ is the primary reactive oxygen species that significantly influences the photocatalytic process [68]. Furthermore, upon the addition of the scavenger EDTA and IPA, it was discerned that the participation of holes and ^•^OH radicals was also apparent due to a slight decrease in efficiency. The prevailing impact of O_2_^−^, as opposed to ^•^OH and holes, is anticipated, given that the electrons generated from the conduction band of g-C_3_N_4_ can directly catalyze the reduction of O_2_ into O_2_^−^, while the holes generated from the valence band of g-C_3_N_4_ cannot directly facilitate the oxidation of H_2_O/OH into ^•^OH [69,70]. As a result, the modification of the band structure of graphitic carbon nitride nanostructures can activate the photocatalyst in oxidation processes and facilitate their use in reactions related to the simultaneous formation of various reactive species, including the removal of chemical and pharmaceutical pollution, water splitting, CO_2_ and nitrogen reduction reactions, and the conversion of hydrocarbon products [71,72].

## 6 Conclusion

The g-C_3_N_4_ and their acid-modified nanostructures were synthesized via thermal polycondensation of urea at varying temperatures and acid-treatment conditions. XRD spectra revealed the presence of melem and its derivatives following processing at a lower temperature of 500 °C, indicating the incomplete transformation of melamine to g-C_3_N_4_. Treatment at higher temperatures and the HCl acid-treatment process (C55 and CHC) led to the formation and expansion of g-C_3_N_4_ networks, corroborated by notable distinctions in peak intensities observed in their FTIR and Raman spectra. Scanning electron microscopy analysis demonstrated a transition from the granular morphology of melamine to the layered structure characteristic of g-C_3_N_4_. Notably, the nanoparticle morphology observed in the CHN sample was attributed to the deconjugation of nanosheets through the highly oxidative HNO_3_ acid-treatment condition. An increase in the BET surface area and porosity of the samples was observed with escalating process temperatures, coinciding with an increase in the bandgap energy. However, despite these trends, the photocatalytic activity for MB reduction under UV and visible light illumination was most pronounced in the C55 and CHC samples prepared at the highest temperature and post-processed situation, respectively. This outcome suggests that factors beyond surface area and bandgap influence the photocatalytic performance. It is proposed that the enhanced photocatalytic activity observed in the C55 and CHC samples may be attributed to reduced recombination of photogenerated charge carriers facilitated by heterojunctions formed between different intermediate phases. These findings underscore the potential of g-C_3_N_4_ and its derivatives as promising photocatalytic materials for water purification applications and acid modification as a cost-effective, simple, and suitable method for the enhancement of catalytic activities of photogenerated charge carriers of CN-based nanostructures in conduction and valence band.

## Claim of interest

The authors claim no conflict of interest.

## Data availability

The data that support the findings of this study are available from the corresponding author upon request.

## CRediT authorship contribution statement

**S.H. Mousavi-Zadeh:** Writing – original draft, Visualization, Validation, Investigation, Data curation, Conceptualization. **R. Poursalehi:** Writing – review & editing, Supervision, Resources, Project administration, Investigation, Data curation, Conceptualization. **A. Yourdkhani:** Writing – review & editing, Supervision, Project administration, Investigation, Conceptualization.

## Declaration of competing interest

The authors declare that they have no known competing financial interests or personal relationships that could have appeared to influence the work reported in this paper.
